# Recurrent soft tissue inflammation, necrotizing fascitis or Sweet syndrome, diagnostic dilemma

**DOI:** 10.1002/ccr3.2445

**Published:** 2019-11-13

**Authors:** Ankit Anand, Teresa Gentile, Hiroshi Kato, Qun Wang

**Affiliations:** ^1^ SUNY Upstate Medical University Syracuse NY USA

**Keywords:** bortezomib, multiple myeloma, necrotizing fasciitis, necrotizing Sweet syndrome, Sweet syndrome

## Abstract

Necrotizing Sweet syndrome is a recently described entity that can pose significant challenges for management. Although necrotizing fasciitis can be rapidly fatal in the absence of prompt surgical management, necrotizing Sweet syndrome may actually be worsened by any surgical intervention. Authors want to emphasize and increase awareness of this rare presentation.

## Graphical abstract

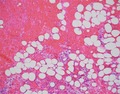

## 1 INTRODUCTION

Sweet syndrome was first described by Robert Douglas Sweet in 1964 as an acute inflammatory skin eruption with fever and leukocytosis.^1^ Pathogenesis of Sweet syndrome is not well characterized although cytokine dysregulation and hypersensitivity reaction in a genetically susceptible individual have been proposed.^2^ Characteristic pathology findings include edema and neutrophilic infiltration of the superficial dermis. Variants like subcutaneous Sweet syndrome have been described with involvement of fat and necrotizing panniculitis.^3^ More recently, acute necrotizing neutrophilic dermatosis was described as a new necrotizing variant of Sweet syndrome, involving the fascia and deep tissues often misdiagnosed as necrotizing fasciitis.^4^ It is imperative to distinguish this new entity from necrotizing fasciitis as management is different.

Patient is a 56‐year‐old Caucasian male with past medical history significant for IgG monoclonal gammopathy for over 15 years and a history of recurrent sinusitis for which his rheumatologist started him on intravenous immunoglobulin. During this time, he describes tender subcutaneous nodules which come and go primarily on his lower extremities, but never needed any medical management. He progressed to multiple myeloma with high‐risk cytogenetics and was treated with lenalidomide, bortezomib, and dexamethasone combination (RVD) for four cycles. Patient achieved a partial response and underwent autologous stem cell transplant with reinfusion of 5.77 million CD34 + cells per kg. Course was complicated by hospital‐acquired pneumonia which responded well to antibiotics. He again developed tender subcutaneous nodules on his upper thighs around day 10 which resolved prior to discharge.

On Day + 32, patient presented to the hospital with 39‐degree fever and erythema/induration of right inguinal area extending to perineum. He was treated with vancomycin and piperacillin‐tazobactam. Blood and fungal cultures were negative, and he was discharged on levofloxacin and doxycycline to a complete 14‐day course.

**Figure 1:**
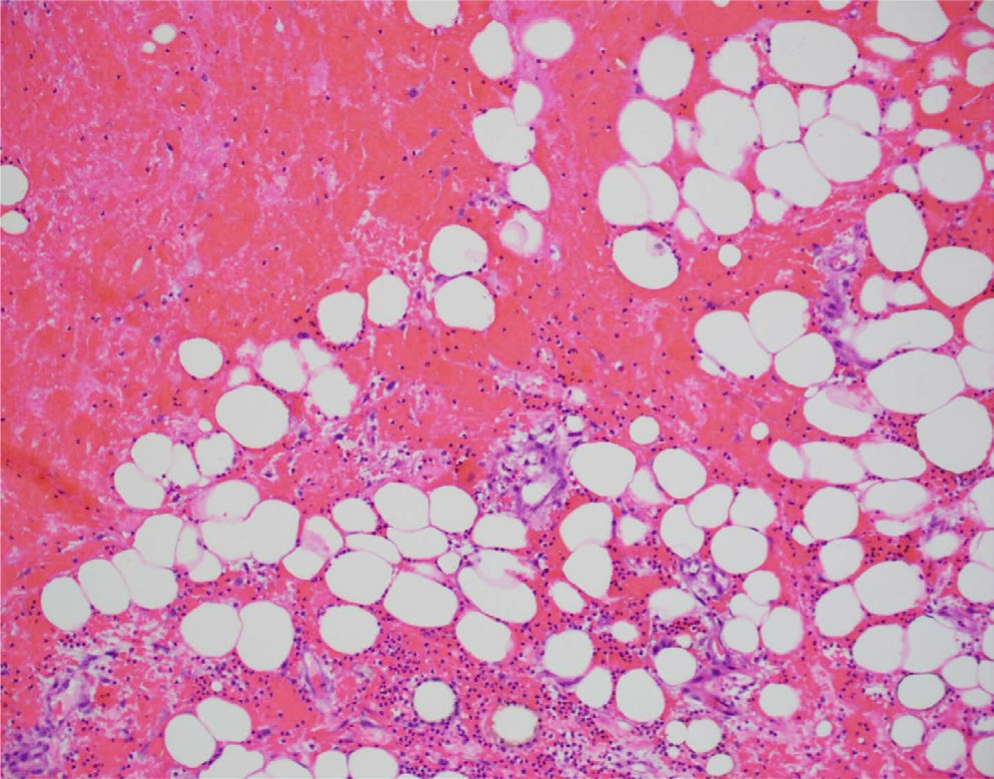
Muscle biopsy showing acute inflammation, necrosis, and hemorrhage

About Day + 60, he was started on maintenance bortezomib given his high‐risk cytogenetics. Shortly after, he again developed increased induration and erythema in right inguinal area. CT imaging was negative for abscess, and doppler was negative for blood clots. Cultures were drawn, and he was started on vancomycin and piperacillin‐tazobactam. Induration and erythema continued to worsen despite antibiotics. Repeat CT imaging revealed worsening stranding in right inguinal region with extension to the anterior lateral and medial right thigh with fluid tracking along the medial right gracilis muscle: concern for necrotizing fasciitis. He continued to spike high‐grade fever; white cell count was 7000 with absolute neutrophil count of 6150. Clindamycin was added and urgent exploration was performed. He underwent wound debridement and was noted to have severely inflamed subcutaneous tissue and fascia with grayish hue. The entire discolored fascia was resected and thereafter three inches of necrotic gracilis muscle was resected. Cultures were drawn, wound vac placed, wound was irrigated and packed. Patient continued to spike fevers with increasing induration and underwent another exploration 48 hours later. There was no necrosis, muscle twitched to electrocautery and no frank infection was noted. Muscle biopsy was done and wound was copiously irrigated and closed. Blood cultures and fungal cultures were negative, patient appeared nontoxic but he continued to spike 39‐degree fever with worsening erythema and induration. MRI revealed enhancement along superficial and deep fascial planes with concern for fat necrosis. Muscle biopsy showed marked acute inflammation, necrosis and hemorrhage as seen in Figure 1. Immunohistochemical stains for CD38 and CD138 were negative, as was the kappa lambda in situ hybridization. Gram stain on the tissue was negative. Wound vac was regularly changed and wound looked healthy with beefy red base and healthy granulation tissue. With continued clinical improvement over next few days, vancomycin and clindamycin were stopped and patient was discharged home to a complete a 35‐day course of piperacillin‐tazobactam. Thereafter, he was put on suppressive penicillin prophylaxis. He continued to get his monthly IVIG in the meantime and maintenance bortezomib was discontinued.

Around Day + 200, he was noted to have worsening paraprotein with a band in gamma region quantified at 3.3 gm/dL consistent with myeloma progression. He received three weekly doses of bortezomib with improvement in his myeloma markers.

**Figure 2:**
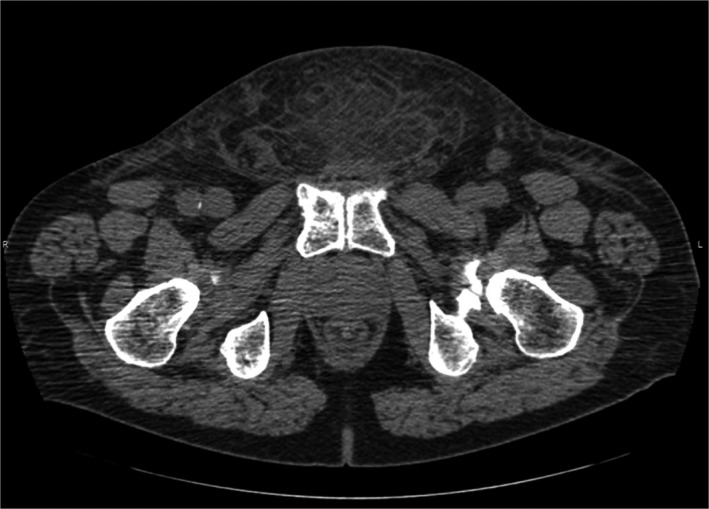
CT showing fat stranding in lower pelvic wall

He presented to hospital on Day + 224 with high‐grade fever, suprapubic lump and scrotal swelling. CT imaging revealed fat stranding in lower pelvic wall but no fluid collection as shown in Figure 2. Cultures were drawn, and he was started on vancomycin, clindamycin, and piperacillin‐tazobactam. Prompt surgical and infectious disease consultations were obtained. No urgent surgical procedure was planned as there was no evidence of true necrotizing fasciitis from the last episode and that his cultures were negative. Scrotal swelling worsened over next few days. CT pelvis revealed thickening of scrotal wall, stranding along the abdominal soft tissues, bilateral reactive inguinal adenopathy and no gas was appreciated. Patient developed left thigh swelling and induration over next few days. MRI left thigh revealed enhancement involving subcutaneous tissue around left thigh musculature. There was mild enhancement of superficial and deep fascial planes, concerning for fasciitis. Aspiration of the suprapubic fullness was performed. This revealed 1 + white blood cells and was negative for any organisms. Cultures later came back negative. Given no improvement with antibiotics so far, he was taken to the operating room for an exploration which revealed healthy tissues and no evidence of necrotizing infection. Another wound vac was placed. Suprapubic tissue was sent for pathology and revealed fat necrosis with acute and chronic inflammation, histiocytic aggregates, but no evidence of plasma cell neoplasm. Fungal and acid‐fast bacilli (AFB) stains were negative. He continued to spike fevers and repeat MRI of left thigh revealed worsening enhancement involving subcutaneous tissues around left thigh musculature along with mild enhancement of the superficial and deep fascial planes compatible with fasciitis.

Given his history of frequent sinus infections, malar rash and an elevated C‐reactive protein (CRP) of 106.1, rheumatology consultation was obtained. Initially thought to be lupus, work up was negative. His original muscle biopsy was re‐reviewed which revealed dense inflammation and differential diagnosis was whether this could be bortezomib‐induced muscle necrosis or necrotizing Sweet syndrome or a yet unidentified condition affecting patients with dysregulated immune system who have been exposed to bortezomib.

Patient was started on high‐dose methylprednisolone 1000 mg daily. Over the next few days, there was improvement in left thigh swelling, scrotal swelling and fever. Antibiotics were discontinued and patient was discharged home on prednisone 1 mg/kg with a slow taper.

Bortezomib was discontinued. While on prednisone taper, his swelling worsened and CT imaging showed diffuse subcutaneous soft tissue stranding compatible with edema/inflammation along the medial and distal posterior aspect of the left thigh and fluid along the fascial planes concerning for fasciitis. He responded to an increase in steroid dose without antibiotics.

## 2 DISCUSSION

Sweet syndrome or acute febrile neutrophilic dermatosis is the prototype of neutrophilic dermatosis; characterized by fever, erythematous papules/plaques, nonvasculitic neutrophilic infiltration of dermis and neutrophilia.^5^ This uncommon disorder is commonly described in association with malignancies, drugs, inflammatory bowel and respiratory diseases.^2^ Around 20% patients with Sweet syndrome have an underlying malignancy, most commonly hematologic malignancy. A thorough search for an underlying neoplasm should be undertaken if not already diagnosed. Sweet syndrome can be diagnosed with fulfillment of both major criteria (abrupt onset of painful plaques/nodules; histopathologic evidence of dense dermal neutrophilic infiltrate in the absence of vasculitis) and at least two of four minor criteria (fever > 38°C; predisposing condition; response to systemic steroids or potassium iodide; leukocytosis and elevated inflammatory markers).^6^ There has been increased incidence of Sweet syndrome recently, likely secondary to the use of growth factor G‐CSF.^7^ Case reports have emerged over the past few years linking the proteasome inhibitor, bortezomib (used in the treatment of multiple myeloma), and Sweet syndrome. At least 10 cases have been reported in literature which have described Sweet syndrome, histiocytoid Sweet syndrome, Sweet‐like lesions with absence of neutrophilic infiltrate and a case of bortezomib‐induced Sweet syndrome in a patient with multiple myeloma that was confirmed by rechallenge 3 years apart.^8^, ^9^, ^10^ Left untreated, spontaneous resolution can happen in many patients over weeks to months, and this is however less likely with malignancy‐associated Sweet syndrome.^11^

Long before first reported case of Sweet syndrome, Hippocrates in 500 BC described necrotizing fasciitis as a potentially fatal and rapidly progressing infection of soft tissues. Necrotizing fasciitis leads to destruction of fascia, fat, and spread of necrosis along the fascia due to its poor blood supply. Due to good blood supply, muscle is usually spared. All soft tissue components can eventually be involved once the infection spreads. Histologically, it is often characterized by necrotic fascia, neutrophilic infiltration, and microorganisms in the tissue specimen.^4^ Prognosis is largely dependent on high index of clinical suspicion and prompt surgical intervention. Despite imaging studies, use of frozen section biopsy and surgical intervention, mortality rates remain high ranging from 6% to 76% in different series.^12^ Attempts to improve outcomes, largely dependent on early identification and prompt surgical intervention are somewhat limited by lack of universally accepted definitive clinical criteria.

Several cases of subcutaneous Sweet syndrome with intense neutrophilic infiltration of the subcutis in the absence of vasculitis have been described. Most of these cases were associated with acute myeloid leukemia or myelodysplastic syndrome and responded to steroids.^13^ A report described two cases of myositis in relation to all trans retinoic acid; however, diagnosis was not confirmed with a biopsy in those patients.^14^ Another report described a patient with spontaneous fasciitis and neutrophilic infiltration of the muscle. All of these patients responded to steroids and none had evidence of myonecrosis.^15^

Blurring the lines further, eight cases of fascial and/or muscle necrosis, so called necrotizing Sweet syndrome were described over the past 5 years. Table 1 shows the reported cases and characteristics of these patients. Six of these eight patients either had an underlying hematologic malignancy or were diagnosed with one immediately after. Three patients had received G‐CSF which is a known inciting factor for Sweet syndrome.^3^, ^4^, ^16^, ^17^, ^18^, ^19^ Kroshinsky et al first reported this unusual presentation in their case series of three patients. One patient had received G‐CSF and bortezomib; however, it is unclear which agent could be the inciting factor. This patient initially had fever which resolved on admission to hospital, he did not have leukocytosis. Further, since the treating physician for this patient had some experience with this unusual presentation, skin biopsy was done and surgical debridement was deferred for concerns of pathergy. Skin biopsy only revealed fat necrosis in this patient. It can only be speculated whether this patient would have had muscle necrosis or not, if he had surgical debridement. It was indeed commendable for the treating physician to avoid surgical debridement and there was significant improvement with steroids.^4^

**Table 1:** Cases of necrotizing Sweet syndrome

Author/year	Age (years)/Sex	Location	Underlying diagnosis	Antecedent factor	Histo pathology	Response with steroid
Kroshinsky et al, 2012	61/M	Antecubital area	CLL	G‐CSF	Fascia and myonecrosis	Yes
Kroshinsky et al, 2012	42/M	Thigh	Burkitt lymphoma	G‐CSF	Fat and myonecrosis	Yes
Kroshinsky et al, 2012	84/M	Arm	Multiple Myeloma	G‐CSF Bortezomib	Fat necrosis	Yes
Nakanishi et al, 2016	73/M	Abdomen	CML, diagnosed later	None	Muscle necrosis	Yes
Minamiyama et al, 2017	54/M	Bilateral arms	MDS		Fascial necrosis	Yes
Otero et al, 2017	24/Trans sex M	Neck/Upper Chest	None		Myonecrosis	Yes along with colchicine and cyclosporine
Lipof et al, 2017	47/M	Hand	None	Hand surgery for Dupuytrens	Myonecrosis	Yes, along with Anakinra, Colchicine
Shen et al, 2017	36/M	Leg	MDS, diagnosed later	None mentioned	Soft tissue necrosis	Yes

The case reported here is likely multifactorial. Patient had a history of recurring nodules with spontaneous resolution prior to treatment for myeloma when he was noted to have MGUS. He then developed the necrotizing lesions, mimicking necrotizing fasciitis, when treated with bortezomib. He did not improve with antibiotics and multiple cultures were negative. He improved with steroids, but on tapering the steroids, the lesions worsened and subsequently improved again with increasing the dose of steroids.

Differentiating necrotizing fasciitis from necrotizing Sweet syndrome is of utmost importance. Although prompt surgical intervention can improve mortality associated with necrotizing fasciitis, surgical intervention can increase morbidity associated with necrotizing Sweet syndrome. This issue is further complicated by the fact that there is no universally accepted definitive clinical criterion for diagnosis of necrotizing fasciitis. Certain features in the history, such as an underlying hematologic malignancy or use of certain drugs may point toward necrotizing Sweet syndrome; however, this cannot conclusively differentiate the two, since two of the eight cases noted herein had no underlying hematologic malignancy or antecedent factors. The diagnosis of necrotizing Sweet syndrome should be considered in any patient suspected of necrotizing fasciitis who fail to isolate any microorganism and fail to respond to antibiotic therapy. A trial of steroids should be considered while covering with antibiotics in such patients.

## CONFLICT OF INTEREST

None declared.

## AUTHOR CONTRIBUTIONS

AA: Participated in clinical care of the patient. Wrote the first draft and submitted the paper. TG: Supervised the Clinical Care of the patient. Reviewed and supervised writing subsequent drafts. HK: Participated in clinical care and reviewed the final draft. QW: Pathologist on the case. Provided Pathological expertise, pathology figures and reviewed the final draft.
